# The role of low Gd concentrations on magnetisation behaviour in rare earth:transition metal alloy films

**DOI:** 10.1038/s41598-020-66595-5

**Published:** 2020-06-17

**Authors:** O. Inyang, A. Rafiq, C. Swindells, S. Ali, D. Atkinson

**Affiliations:** 1 Department of Physics, Durham University, Durham, UK; 2 Department of Physics, Government College University, Lahore, 54000 Pakistan; 3 Department of Physics, Riphah International University, Lahore, Pakistan

**Keywords:** Nanoscience and technology, Physics

## Abstract

The magnetisation reversal behaviour as a function of composition was studied in low rare earth concentration alloys. 30 nm thick rare earth:transition-metal films of composition Gd_*x*_Co_100−*x*_, Gd_*x*_Fe_100−*x*_ and Gd_*x*_(Co_50_Fe_50_)_100−*x*_ were prepared by magnetron sputtering, where *x* ranged from 4 to 13 atomic%. Magnetisation behaviour was studied using MOKE and Hall hysteresis measurements. The magnetic reversal behaviour as a function of Gd content is strongly dependent on the transition metal. With increasing Gd content the film structure transitions from crystalline to amorphous and the saturation magnetisation decreases linearly. For GdCo, the reversal field, *H*_*c*_, increases by less than a factor of two with Gd doping of 11%, while for Fe, the coercivity falls by a factor of ten with 8% Gd. This may be attributed to changes in the crystalline morphology. GdCoFe shows a similar trend with Gd doping for the in-plane reversal field to that of GdFe. With 13% Gd in Fe there is evidence indicating the presence of a weak perpendicular magnetic anisotropy, PMA. With Gd doping the anomalous Hall resistivity of Co, Fe and CoFe increases significantly with the largest increase observed for GdCoFe.

## Introduction

For applications of magnetic materials the processes of magnetisation reversal are often critical for functional performance. Magnetic field driven reversal is most common, but more recently both spin transfer torque (STT) and spin-orbit torque (SOT) mechanisms, driven by current flow, have been developed in spintronics^1–6^. In the development of magnetic materials for such spintronic applications, understanding and controlling the magnetisation behaviour for field, STT and SOT driven reversal mechanisms is therefore necessary. Research has shown that the SOT arises from spin-orbit interactions that can generate spin current to switch the magnetisation in systems based on heavy metal/magnetic bilayers^3,7^.The search for materials and material combinations exhibiting a large SOT is actively investigated, with W, Ta, and Pt among the heavy metals (HM) that show large SOT^3,7,8^. The quest for alternative magnetic materials has been extended from ferromagnets (FM) to ferrimagnetic^9–14^ and antiferromagnetic materials (AFM)^15,16^, where the latter are advantageous for fast manipulation, no stray fields and low-power consumption^17^. Ferrimagnetic materials are attractive as they combine some of the advantages of both ferro and antiferromagnetic materials. These materials have also recently received renewed attention for a range of applications including all optical ultrafast magnetisation switching^18^, multilevel current induced switching^19^, fast current driven domain walls^20^ and skyrmions for device applications^20,21^, and THz generation^22^.

Research on the rare-earth:transition-metals (RE:TM) ferrimagnets has a long history and is currently resurgent with their prospects for SOT devices^10–12,14,17^. In these studies the transition metal is typically a CoFe alloy. RE:TM ferrimagnetic systems with around 20–25 atomic percent (at%) RE are typically amorphous and most current interest is linked with a bulk perpendicular magnetic anisotropy (PMA) that is often, but not always, obtained during film deposition. The anisotropy in RE:TM systems is of fundamental importance, with initial interest in the formation of amorphous RE:TM thin films by co-evaporation of the constituent elements by Orehotsky and Schroeder^23^, while Chaudhari *et al*. and Rhyne *et al*. later prepared amorphous RE-TM alloys by sputter deposition^24,25^.

Investigations have shown the importance of the RE concentration on the magnetic properties^26^, although such studies have focused on RE content above 20 at%, rather than the low doping regime, which is the subject of this report, where the onset of the effects resulting from RE doping are presented.

Previous fundamental studies have tended to focus on RE elements in combination with a single TM ferromagnetic element, while SOT studies have often focused on RE:CoFe alloys, which opens the question as to the role of the specific TM elements in such TM alloys. The presence of a uniaxial magnetic anisotropy is the most common feature of the amorphous RE:TM thin films. The orientation and strength of this anisotropy depends upon temperature, composition, and details of the preparation^27^. For instance, Gd-Co films prepared by vapor-deposition were shown to be magnetically anisotropic with an easy axis in the film plane^27–30^, while vapor-deposited films of other RE:TM alloys display perpendicular anisotropy^31,32^. Studying the early stage evolution of the in-plane and out-of-plane magnetisation behaviour in RE:TM alloys with low RE concentration may therefore contribute to understanding of the development of the anisotropy in these alloys.

Here, a study of the initial changes in the magnetisation behaviour and the development of anisotropy in RE:TM systems in the low RE doping regime is presented. The RE:TM alloys used in this research are GdCo, GdFe and Gd(Co_50_Fe_50_), which are among the well-known RE:TM^33,34^ alloys. In these alloys, the RE 4f magnetic moments and TM 3d moments interact via a negative exchange interaction giving rise to ferrimagnetic order. Since this work reports the influence of RE concentration on the magnetisation reversal behaviour of GdCo and GdFe it also aims to understand the role of these transition metal species in governing the magnetisation behaviour of GdCoFe alloys.

## Experimental Methods

Rare earth:transition metal alloy samples were prepared by co-sputtering from two targets in a high vacuum base-pressure chamber using d.c. magnetron sputtering with an Ar gas deposition pressure of 10^−3^ torr. Here the RE element was Gd and the transition metals were Co, Fe and Co_50_Fe_50_. The alloy films were deposited onto a tantalum buffer layer of thickness 5 nm on to oxidized Si substrates. The RE:TM films were 30 nm thick and deposited at rates between 0.04–0.36 nms^−1^, as determined using a quartz crystal monitor during deposition and confirmed by the thickness from X-ray reflectivity analysis. A 1 nm Ta capping layer was deposited to prevent oxidation. The Gd concentration, $x$, was varied with the following concentrations, *x* = 4, 6, 8, 11 and 13 at%. This low Gd content regime was investigated to understand the influence of the RE at low concentrations upon the magnetisation behaviour of the TM thin-films. The atomic compositions were determined for a number of samples using energy dispersive X-ray spectroscopy, EDX, in a scanning electron microscope. The variation of the density of the RE:TM alloy through the film thickness was studied as a guide to the uniformity of the distribution of the Gd within the alloy layer. This was undertaken on separate Pt/GdCo/Pt samples, prepared in the same system, using X-ray reflectivity measurements at the Gd *L*_3_-edge carried out on beam-line 4-ID-D of the Advanced Photon Source at Argonne National Laboratory, with analysis undertaken using the GenX code^35^. The crystal structure for selected samples was studied using X-ray diffraction, XRD, with Cu ${K}_{\alpha }$ radiation. Room temperature saturation magnetisation, M_s_, was obtained using a superconducting quantum interference device (SQUID) magnetometry and the magnetisation reversal behaviour was measured using in-plane longitudinal magneto-optical Kerr effect (MOKE) magnetometry. Hall effect measurements were used to investigate the anomalous Hall resistivity and the out-of-plane magnetisation behaviour, the latter, in combination with longitudinal MOKE, giving insight into the anisotropy. The in-plane reversal behaviour was characterised by the coercivity, H_c_, which was extracted from the MOKE hysteresis loops. Sample preparation and all measurements were performed at room temperature.

## Results

**Figure 1:**
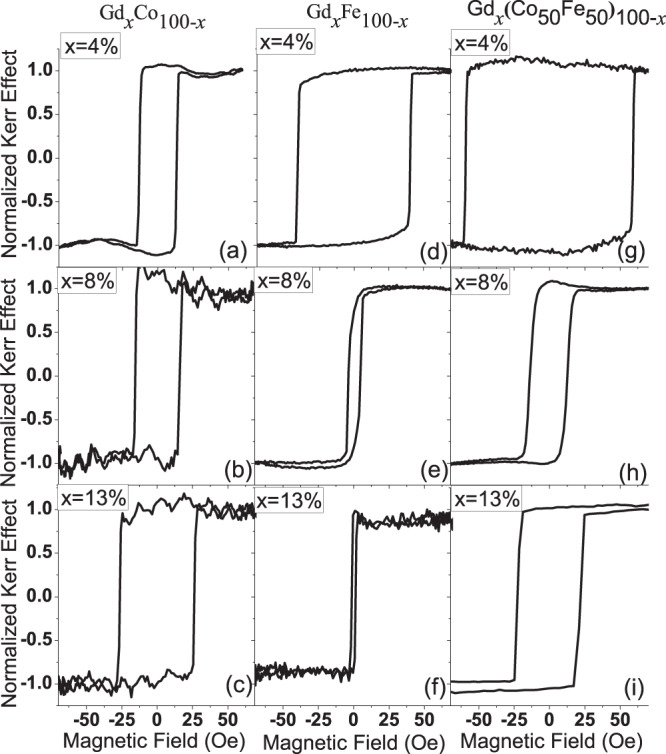
Magnetic hysteresis loops from longitudinal MOKE measurements of Ta(5 nm)/Gd_*x*_TM_100−*x*_(30 nm)/Ta(1 nm) thin films where TM is Co, Fe and Co_50_Fe_50_ as labelled.

**Figure 2:**
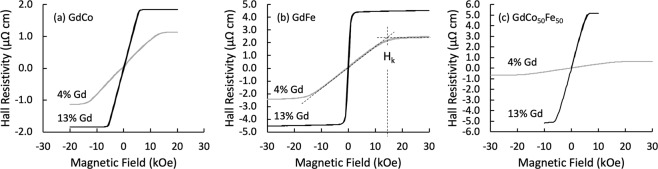
The anomalous Hall effect as a function of the out-of-plane magnetic field for 30 nm thick RE:TM films. The smaller curves represent 4 atomic percent Gd and the larger curves 13 atomic percent Gd. The dashed lines in on the curve in (**b**) indicates the estimation of the anisotropy field, H_*k*_ Note different scales for Hall resistivity.

**Figure 3:**
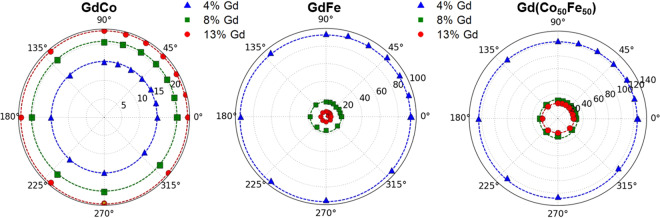
Angular dependence of coercivity, *H*_*c*_, of RE:TM alloys with low Gd doping in (**a**) GdCo (**b**) GdFe and (**c**) Gd Co_50_Fe_50_.

Examples of the magnetic hysteresis measured with longitudinal MOKE are shown in Fig. 1 for different atomic concentrations of Gd, up to 13%, in Co, Fe and Co_50_Fe_50_ thin films. For all of the films investigated the in-plane remanence was close to 100%, indicating that the magnetisation is in-plane. This is supported by the anomalous Hall effect measurements, see Fig. 2, which show near anhysteretic behaviour that saturates at high field, indicative of magnetisation change along a hard magnetic axis. The longitudinal MOKE hysteresis loops all show high remanent magnetisation, suggesting an in-plane anisotropy easy axis, however, in all cases hysteresis loops measured as a function of the in-plane field angle show isotropic in-plane behaviour characterised by high remanence ratio and no significant angular dependence of the coercivity, as summarised in Fig. 3. Here, as discussed later, the crystallographic structure transitions from polycrystalline assemblages to amorphous across the Gd concentration studied and both of these structures having no long-range structural ordering that would give rise to global magnetic anisotropy. This may explain the resultant isotropic in-plane magnetisation behaviour and high remanence ratio observed along all in-plane axis, which suggests a field-induced magnetic easy axis aligned in the direction of the magnetic field on the application of the field. Taken together, this data shows a clear in-plane easy magnetisation for the RE:TM alloys in the low Gd concentration regime for GdCo, GdFe and GdCoFe alloys.

**Figure 4:**
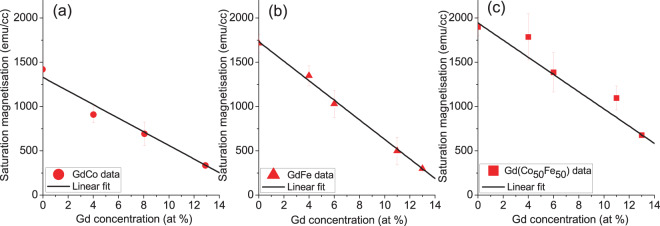
The saturation magnetisation, *M*_*s*_, of 30 nm thick films of Ta(5 nm)/Gd_100−*x*_TM_*x*_/Ta(1 nm). The solid lines are the linear fit to the data. Data for zero at % of Gd obtained from reference^37,38^.

In nanoscale magnetic films and structures, self-demagnetising fields play a significant role in determining the magnetisation behaviour and hence knowledge of the saturation magnetisation of the material is needed in order to understand any compositional dependence of the magnetisation behaviour. Figure 4 shows the saturation magnetisation as a function of Gd concentration in Co, Fe and CoFe alloys. With increasing Gd doping, the saturation magnetisation, *M*_*s*_, decreases linearly for the Co, Fe and CoFe alloys. For the maximum doping here of 13 at% Gd, *M*_*s*_ falls to 334 ± 28 emu/cc, 295 ± 7 emu/cc and 678 ± 5 emu/cc for GdCo, GdFe and GdCoFe respectively, which are comparable to values reported in the literature^26,30,36^. As a result of the RE:TM antiferromagnetic coupling, the alloy values are lower than for pure Co, Fe and CoFe transition metals which are 1422 ± 7 emu/cc, 1714 ± 7 emu/cc and 1900 ± 5 emu/cc respectively^37,38^.

## Discussion

Both the in-plane magnetisation reversal behaviour, from MOKE, and the out-of plane magnetisation behaviour, from Hall measurements, as a function of Gd doping depend critically on the transition metal ferromagnet in the alloy. It should also be noted that for the low levels of Gd doping studied here, any ferrimagnetic compensation point would occur well below room temperature and hence is not a factor in the behaviour observed in this study at room temperature.

**Figure 5:**
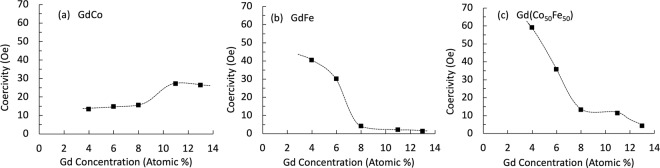
The in-plane coercivity, *H*_*c*_, of 30 nm thick films of Ta(5 nm)/Gd_100−*x*_TM_*x*_/Ta(1 nm). The dashed lines are guides to the eye.

Figure 5 summarises the in-plane magnetisation reversal behaviour by showing the coercivity, ${H}_{c}$, as a function of Gd concentration for GdCo, GdFe and GdCoFe alloy thin-films. The trends in the ${H}_{c}$ behaviour with Gd doping in Co and in Fe are quite different. For GdCo alloys, ${H}_{c}$ is nearly constant up to 8 at%, then ${H}_{c}$ doubles in one step and remains at this level up to 13 at% Gd doping, the highest doping studied here. In contrast, the introduction of Gd doping into Fe leads to a rapid reduction of ${H}_{c}$, resulting in an order of magnitude increase with the addition of only 8 at% Gd. Beyond 8% Gd doping, ${H}_{c}$ is very low and nearly constant with higher Gd content, which agrees with previous work on GdFe thin films^39^. Now, since ${M}_{s}$ decreases linearly with increasing Gd doping the self-demagnetising field will also reduced linearly, which does not explain the trends observed for the in-plane ${H}_{c}$. In a simple model, a linear reduction in the demagnetising field, due to lower ${M}_{s}$, would linearly increase the values of ${H}_{c}$, which is not observed for any alloys here.

For Co and Fe the out-of-plane magnetisation behaviour with increasing Gd doping shows similar trends in terms of the saturation field, but with different magnitudes of change. For both Co and Fe, increasing Gd doping from 4 to 13 at% significantly reduces the out-of-plane saturation field from 13 kOe down to 6 kOe for GdCo and from about 14 kOe to 1 kOe for GdFe. In contrast to the in-plane magnetisation behaviour, the out-of-plane magnetisation behaviour is affected by the reduction of *M*_*s*_ due to Gd doping, because it leads to a lower perpendicular demagnetising field, which reduces the out-of-plane saturation magnetic field, as observed for all alloys here. However, whilst the magnitude of the change in the saturation field of the CoGd alloy is largely explained by the demagnetising field, the out-of-plane saturating field for the FeGd alloy is signifcantly lower than that expected simply from the reduction of the out-of-plane demagnetisation field. Hence other factors must also be involved.

**Figure 6:**
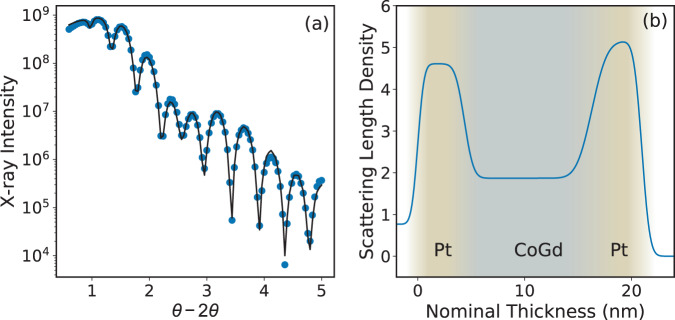
(**a**) X-ray reflectivity data (symbols) and the best fitting simulation (solid line) for a Pt/GdCo/Pt trilayer structure. (**b**) The scattering length density through the trilayer derived from the X-ray reflectivity analysis.

To understand the doping dependent changes of magnetisation behaviour, it is therefore useful to combine both the structural and magnetic effects associated with RE doping of TM ferromagnets. Considering first the uniformity of the Gd distribution, Fig. 6(a) shows an example of the specular X-ray reflectivity data and the simulation for the best-fitting model for the Pt/GdCo/Pt sample. Derived from this model, Fig. 6(b) shows the variation of the scatter length density, related to the electron density, through the sample thickness. The upper and lower Pt layers are clear with higher scattering density, while the GdCo alloy layer displays a constant electron density through the thickness of the film. This indicates the alloy composition, and hence the Gd distribution, is uniform through the alloy thickness. The transition between the two regions of constant electron density is indicative of the interface width between the CoGd and Pt layers. The addition of RE elements to transition metals changes the microstructure from the TM crystalline state to an amorphous alloy structure. The transition occurs over a small doping range with a reduction of the crystallite size until no crystalline signature is detected. Mapping of this transition in the literature is limited and the doping level for the onset of the amorphous state appears sensitive to the elements involved and may also depend on the details of the deposition conditions. A previous detailed XRD study of GdFe has shown a transitional microstructure at 13% and a full amorphous state for 16% Gd^39^, while for GdCo, an amorphous state was noted for all Gd concentrations down to 5 atomic percent^30^ and for GdNi the structure was crystalline for 5% and amorphous for 15% Gd respectively^40^. The RE:TM alloys with around 23% RE are known to commonly exhibit perpendicular magnetic anisotropy, the origin of which has been the subject of debate with experimental evidence indicating bond-orientational anisotropy gives rise to this bulk PMA^41^. It is also long established that the Gd moment couples antiferromagnetically with the TM moment^42^, which is mediated by an indirect mechanism that combines positive *f* − *d* and direct *d* − *d* exchange interactions within the system^43^.

**Figure 7:**
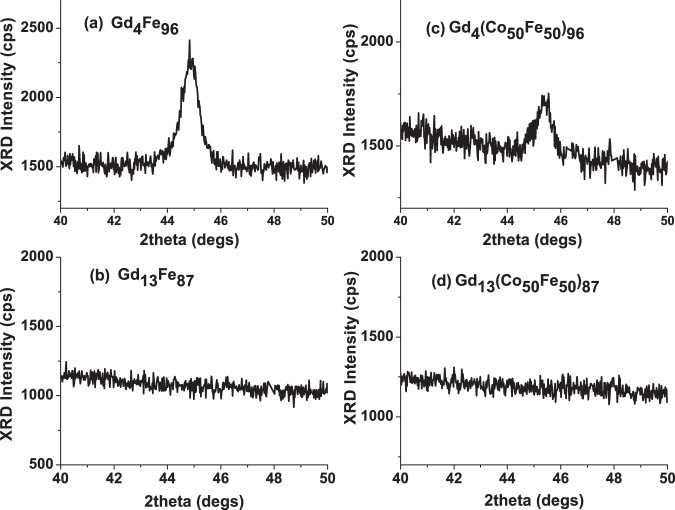
X-ray diffraction profiles of (**a**) Gd_4_Fe_96_ (**b**) Gd_13_Fe_87_ (**c**) Gd_4_(Co_50_Fe_50_)_96_ and (**d**) Gd_13_(Co_50_Fe_50_)_87_.

**Figure 8:**
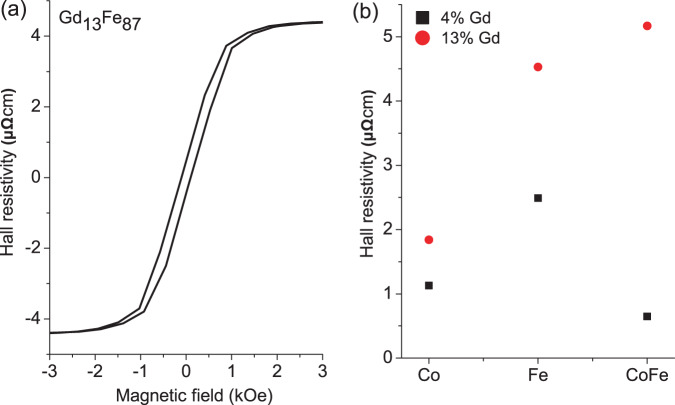
(**a**) Hall effect measurement for Ta(5 nm)/Gd_13_Fe_87_(30 nm)/Ta(1 nm) thin film. (**b**) Hall resistivity as a function of Gd concentration for Co, Fe and Co_50_Fe_50_.

Considering the microstructure here, XRD shows that for GdFe a crystalline peak is present with 4% Gd, but there is no evidence of crystallinity for 13% Gd, see Fig. 7(a,b). The large reduction of the in-plane ${H}_{c}$ observed for the GdFe series is attributed to an initial reduction of the BCC crystallite size and the transition to an amorphous structure, once this structure is established the magnetisation behaviour may vary little, hence the uniform low ${H}_{c}$ established beyond 10% Gd doping. For the GdCo system the change of in-plane ${H}_{c}$ shows a small step between 8 and 11% Gd and is effectively constant beyond 11%, this step change may be linked with the transition from the polycrystalline HCP Co film structure to an amorphous alloy state. Similar behaviour has been seen elsewhere, where the amorphous state was noted in e-beam evaporated GdCo around 5% Gd^30^. With increasing Gd doping, the antiferromagnetic alignment of the RE and TM moments linearly reduces the net magnetisation, as shown in Fig. 4. This will consequently reduce the out-of-plane demagnetising field and hence reduce the out-of-plane saturation field, which is observed. Comparing the out-of-plane saturation fields of GdFe and GdCo it is clear that whilst both alloys show a reduced saturation field with increasing Gd content the effect is significantly larger for GdFe, even though the changes in saturation magnetisation with Gd doping are comparable, as evidenced here and elsewhere^39,44^. It is therefore suggested that the out-of-plane behaviour is also influenced by the first stages of the development of a bulk perpendicular magnetic anisotropy, which forms the basis for much research activity in higher Gd content alloys. This is particularly the case for the GdFe alloy, and there is direct evidence for an out-of-plane component of magnetisation in the hysteresis of the Hall measurement of the GdFe film with 13% Gd, which shows a small perpendicular remanent magnetisation, see Fig. 8a. This indicates the presence of a weak PMA. This PMA will act against the self-demagnetising field and further reduce the field required to rotate the magnetisation out-of-plane. In the case of CoGd this hysteresis is not observed and hence does not help to counteract the out-of-plane self-demagnetisation. Taken together, the reduction in the the net magnetic moment with Gd doping of Co and Fe and the onset of a weak PMA in GdFe explains the observed out-of-plane magnetisation behaviour.

With similar Gd doping dependence on the saturation magnetisation, but clearly different effects of Gd doping on the magnetisation reversal behaviour of Co and Fe films, it is interesting to understand the role of the TM element in determining the magnetisation behaviour in RE:TM ternary alloys. For Co_50_Fe_50_ the dependence of the in-plane ${H}_{c}$ on Gd doping is comparable with that of GdFe rather than GdCo, which indicates an important role for the Fe in the alloy. For GdCo_50_Fe_50_ the in-plane ${H}_{c}$ drops rapidly up to 8% Gd and varies little with further Gd doping. This can also be linked to microstructural changes with Gd doping, as this GdCo_50_Fe_50_ alloy composition has an initial BCC crystal structure in common with Fe and shows a similar change to an amorphous structure in the XRD, see Fig. 7. Interestingly, the out-of-plane magnetisation behaviour of GdCoFe, obtained from the Hall measurements, also shows a reduction of the out-of-plane saturation field from 17 kOe down to 5.4 kOe for Gd from 4 to 13%. The magnitude of this change is between that for GdCo and GdFe. It is also noted from the Hall measurements that there is no out-of-plane remanence for the highest Gd doping of CoFe alloy, indicating no onset of a PMA for low Gd doping of GdCo_50_Fe_50_.

Finally, even low doping with Gd has a significant effect on the magnitude of the anomalous Hall effect in these RE:TM alloys. Figure 8b compares the Hall resistivities of Co, Fe and CoFe thin-films with 4% and 13% Gd doping. The origin of the anomalous Hall effect is of course quantum mechanical in nature, but can be understood in semi-classical terms via three contributions^45^, associated with intrinsic deflections, side jump scattering and skew scattering of the conduction electrons. Here changes of microstructure, the addition of a RE metal and changes in magnetisation may modify all three mechanisms and hence change the anomalous Hall effect. The largest anomalous Hall resistivity was observed for 13% Gd doping of GdCo_50_Fe_50_.

## Conclusion

In conclusion, the influence of RE concentration on the magnetisation reversal behaviour of Gd:Co, Gd:Fe and Gd:CoFe alloys was studied in order to understand the role of the RE in the regime of low doping and the choice of the transition metal. The results show that both the in-plane and out-of-plane magnetisation reversal behaviour as a function of Gd content are strongly dependent on the transition metals in the alloy. As the Gd content increases, the thin films transition from a crystalline to an amorphous structure at a few percent Gd. The Gd dependence of the in-plane magnetisation reversal behaviour is characterised by the coercivity, ${H}_{c}$, which increases by less than a factor of two with Gd doping of 11% for GdCo, while in contrast, Gd doping of Fe reduces the coercivity by a factor of ten with 8% Gd. This is attributed to changes from crystalline to amorphous morphology in both cases. Interestingly, GdCoFe shows a similar trend with Gd doping for the in-plane ${H}_{c}$ to that of GdFe. At 13% Gd in Fe there is also evidence suggesting the presence of a weak PMA. With additional Gd doping the anomalous Hall resistivity of Co, Fe and CoFe increases significantly, with the largest increase observed for the GdCoFe alloy. The results reported here provide a more detailed understanding of the role of the initial RE doping on both the structural and magnetic properties of RE:TM ferrimagnetic alloys, providing greater understanding of the physics to underpin future spintronic devices.
